# Legislative compliance in coordinated care transitions: a mixed-method study of healthcare professionals' documentation and older adults' experiences

**DOI:** 10.1080/17482631.2026.2615154

**Published:** 2026-01-15

**Authors:** Emelie Ingvarsson, Heidi Hagerman, Catharina Lindberg, Mirjam Ekstedt, Kristina Schildmeijer

**Affiliations:** a Department of Health and Caring Sciences, Linnaeus University, Kalmar/Växjö, Sweden; b Department of Research, Region Kalmar County, Kalmar, Sweden; c Department of Learning, Informatics, Management and Ethics, Karolinska Institutet, Stockholm, Sweden

**Keywords:** Coordinated care transition, discharge, mixed-method, older adults, patient involvement qualitative

## Abstract

**Purpose:**

This study aimed to explore and describe how coordinated care transitions aligned with legislation when older adults are discharged from in-patient care to their homes.

**Methods:**

A mixed-method (QUAL + qual) design was used. The core data component (QUAL) consisted of copies of 15 older adults' healthcare and social care records. The supplementary data component (qual) encompassed individual interviews. All data related to the same older adults, whose coordinated care transitions took place between January to June 2022. The analytical procedure followed a deductive thematic analysis.

**Results:**

Findings showed that individual care plans were often missing or inadequately documented. Documentation of older adults' participation was frequently poor and inconsistent, with many decisions made without their input. However, some documents and interviews indicated that older adults had genuinely participated. The discrepancy between documented procedures and actual experiences reveals significant variability in older adults' inclusion.

**Conclusion:**

This study highlights the frequent exclusion of older adults from coordinated care transition process and deficiencies in documentation. The findings underscore the urgent need for standardized and inclusive documentation practices, as well as improved communication strategies, to ensure more person-centred care transitions, in which older adults are genuinely involved and well-informed about their care transitions.

## Background

Worldwide, there is a recognised need to shift from a model in which patients passively receive care to one in which they actively participate in co-creating their care. This shift is particularly crucial for addressing the long-term healthcare challenges posed by the growing number of older adults with multiple chronic conditions (Swedish Government, 2020). Older adults, aged 65 years or older (National Board of Health and Welfare, 2022), often require continuous healthcare services and support from multiple healthcare providers (Kuluski et al., 2017; World Health Organisation, 2015). They commonly need to transfer between different care settings and levels of care, for instance from in-patient care to home (Coleman & Boult, 2003). This is because healthcare systems are not yet designed to adequately address the often complex and chronic needs of older adults (Joo, 2023). As many older adults often return home upon discharge, a coordinated care transition is required to ensure continuity and safety (Liljas et al., 2024).

Care transitions pose significant challenges, especially for older adults with chronic diseases, due to their heightened vulnerability (Cline, 2016; Laugland, 2015). The transition from hospital to home is particularly risky because of the potential for adverse events. For the individual, daily life may be impacted post-discharge due to unmet needs, medication concerns, or communication gaps (Andreasen et al., 2015). Problems in a transition can also lead to prolonged periods of care or even death (Schildmeijer et al., 2018), for instance due to continuity gaps, poor communication, and inadequate coordination among care providers and older adults (Beer et al., 2014; Cline, 2016; Kneck et al., 2019; Schoenborn et al., 2013). Patients consider these issues as barriers to achieving optimal discharge processes (Luo et al., 2024). Vogel et al. (2024) found similar results, emphasising the need for improved communication and patient involvement before discharge.

Previous studies indicate crucial gaps in care transitions from in-patient care to the patient's home, with healthcare professionals' (HCPs) often focusing more on care coordination than on involving older adults (Bångsbo et al., 2017; Cam et al., 2023). Older adults have reported feeling unheard and having their concerns or priorities overlooked by healthcare providers during transitions (Liebzeit et al., 2023). HCPs recognise the necessity of involving the patient in the discharge process and prioritising their perspective to achieve an optimal discharge process (Yeh et al., 2024), supported by the patient's views (Luo et al., 2024). However, it has been found that the patient's perspective on participation is commonly absent in HCPs documentation within medical records (Hurtig et al., 2025). Moreover, previous research highlights the importance of a greater focus on the needs reported by older adults in care transitions (Liebzeit et al., 2021). It also shows that older adults want to be involved in their discharge (Gabrielsson-Järhult & Nilsen, 2016), in discharge planning (Schjødt et al., 2022), and in decision-making (Jobe et al., 2019) to various degrees (Nilsen et al., 2022). However, participation in care planning was seen as a key aspect of participation by both patients and HCPs, who concurred that it was essential for patients to gain knowledge and engage in reciprocal communication to improve their participation (Hurtig et al., 2025).

There is increasing recognition, both globally (World Health Organisation, 2017) and nationally in Sweden, of the need to enhance patient involvement and adopt a more person-centred approach to ensure high-quality and equitable care (Swedish Government, 2020; SFS, 2014:821). A person-centred approach, which acknowledges the patient's individual needs, preferences, and resources in healthcare delivery (Swedish Government, 2020) has been shown to improve the discharge process (Marsall et al., 2024; Ulin et al., 2016). Marsall et al. (2024) conclude that high-quality discharge processes can mitigate patient safety risks and improve post-discharge health status at home. Healthcare organisational models vary across countries, and there is no single standardised approach for coordinating and delivering care. This variation results in localised adaptations based on local conditions (Wodchis et al., 2015). In Sweden, the care transition from in-patient care to home is regulated at the national level. This legislative framework emphasises professional cooperation between healthcare providers and the active participation of older adults in creating individual care plans when continued healthcare and social care support is needed after discharge (SFS, 2017:612). Additionally, Swedish legislation underscores older adults' right to participate in planning and decision-making, and the importance of their possibility to exercise authority over their own care decisions (SFS, 2014:821; SFS, 2001:453; SFS, 2017:30).

Coordinated care transitions play a pivotal role in ensuring seamless and effective healthcare delivery. Inadequate coordination and insufficient involvement of older adults during these transitions can result in unavoidable and negative outcomes for the individuals. It is vital to understand how care transitions align with legislation, which often emphasises the importance of patient participation in healthcare decisions. Older adults want and deserve active involvement in their care transitions, and compliance with legislation is essential to ensure that patient rights are upheld throughout such transitions. Hence, this study aims to explore and describe how coordinated care transitions align with legislation and how compliance with legislation concerning patient participation is reported and experienced when older adults are discharged from in-patient care to home. To address this aim, the following research questions have been formulated:

RQ 1. How do individual plans align with legislative requirements?

RQ 2. How is patient participation reported and experienced when older adults are discharged from in-patient care to home?

RQ 3. How do older adults' experiences of participation align with healthcare professionals' reported data?

## Method

### Study design

This study employed a qualitative driven simultaneous mixed-method (QUAL + qual) design (Morse, 2010), chosen because mixed-methods offer methodological principles for managing increasingly complex research problems and enable the integration of multiple qualitative data sources. This integration was necessary to address the specific research questions (RQs 1–3) and to achieve the overall aim of the study. The study was qualitatively driven with an inductive theoretical orientation, aligned with the exploratory and descriptive aim. Consequently, the design comprised a core qualitative data component (QUAL) supplemented by an additional qualitative component (qual) (Morse & Niehaus, 2016).

The analytical procedure was guided by the principles of a QUAL + qual design (Morse, 2010; Morse & Cheek, 2014; Morse & Niehaus, 2016) and followed a theoretical thematic analysis (Braun & Clarke, 2006), i.e. deductively driven with relevant legislative frameworks informing the analytical procedure (Braun & Clarke, 2006).

The core data component (QUAL) included copies of older adults' healthcare and social care records obtained from various healthcare providers, used to address RQs 1–3. The supplementary data component (qual) comprised individual interviews with older adults, contributing data relevant to RQs 2-3. This supplementary data component provided additional insights and perspectives within the context of the core component data (Morse, 2010), and the two datasets were integrated during the analysis, at the *analytical point of interface* (RQ3), and incorporated in the narrative presentation of the core results, at the *results point of interface* (RQ2), in line with principles for QUAL + qual designs (Morse & Cheek, 2014; Morse & Niehaus, 2016).

### Setting

The healthcare system in Sweden is mainly tax-funded, with a high degree of decentralisation (Anell & Glenngård, 2012). The responsibility for providing healthcare and social care services is distributed across 21 regions and 290 municipalities (Bergmark & Minas, 2016; SOU 2018:39, 2018). Regions are responsible for the financing and delivery of in-patient care, specialist care and primary care, whereas municipalities have the responsibility for delivering home healthcare and social care services in older adults' homes (Anell & Glenngård, 2012). The care transition from in-patient care to a patient's home is regulated at the national level, although local adaptations are made by regions and municipalities to coordinate discharges based on local conditions (SFS, 2017:612). This study was conducted in southern Sweden, in a region with nearly 250,000 citizens. The area is served by one county hospital and two smaller hospitals. It has one of the oldest populations in Sweden, and an estimated 80% of citizens live in urban areas.

### Recruitment, participants and data components

The study used purposive sampling (Patton, 2015) with a criterion approach (Polit & Beck, 2021). The inclusion criteria were age 65 years or older, communicating in Swedish, having at least one chronic disease (e.g., heart failure, chronic obstructive pulmonary disease, stroke or diabetes), and having healthcare and social care services coordinated between healthcare providers upon being discharged from in-patient care to home. Older adults diagnosed with general cognitive impairment or dementia were excluded. HCPs working with coordinating care transitions at hospital wards or in the municipality assisted with the initial recruitment of older adults. They informed older adults about the study and asked about their interest to participate. If a potential participant expressed interest, the HCP provided the first author with their contact details, who then initiated contact. We used a multi-step process in which the older adults were repeatedly informed about the study and the fact that participation was voluntary and given the possibility to ask questions and the opportunity to withdraw participation at any time. Twenty-two older adults initially agreed to participate. However, when EI made contact by telephone, five of these older adults withdrew their consent due to changing their mind (*n* = 2), illness (*n* = 2) or being asked not to participate by close relatives (*n* = 1). Additionally, two older adults only participated in the interviews, due to their own choice (*n* = 1) or not having passed the confidentiality check (*n* = 1), meaning that data, which were considered sensitive personal data, could not be disclosed for confidentiality reasons or the risk of harm or detriment to the individual or people close to them. In total, 15 older adults, five men and ten women, aged between 65 and 92 years (mean = 81) participated.

*The core data component* (QUAL) consisted of copies of the older adults' healthcare and social care records (see appendix 1 for an overview) originating from in-patient care, primary care, municipal healthcare, and social care services. The documents touched upon the care transitions from the perspective of the various HCPs involved, such as nurses, physiotherapists and occupational therapists, social care service managers, physicians and home help services. The core component dataset encompassed 28 different types of documents, ranging from five to 17 documents per person (mean = 9), and totalling approximately 274 pages of text, between nine and 33 pages per person (mean = 18).

*The supplemental data component* (qual) consisted of individual interviews with the same older adults to whom the documents pertained. The interviews were conducted simultaneously, as part of data collection in another study by the same research team (Ingvarsson et al., 2024). The interviews encompassed 238 pages of transcribed text in total, ranging from 10 to 24 pages per person (mean = 16). The interviews lasted between 22 and 58 minutes, with an average duration of 35 minutes.

### Collection of data components

Each older adult decided the time and place for their interview, with four conducted by phone and eleven in participants homes, all between January 2022 and June 2022. Each interview began with an open-ended question to encourage the participant to talk about their discharge experiences freely. This was followed by questions about the planning during and after hospitalisation and decision-making process ahead of their discharge, along with probing questions for further elaboration or examples. All interviews were audio-recorded, transcribed verbatim and checked for accuracy against original audio recordings. The process of obtaining hard copies of the documents from each healthcare provider was carried out in parallel with the interviews and completed in March 2023, when all hard copies had been handed over to EI. Data collection was conducted by EI.

### Analytical procedure and integration of datasets

**Figure 1.**
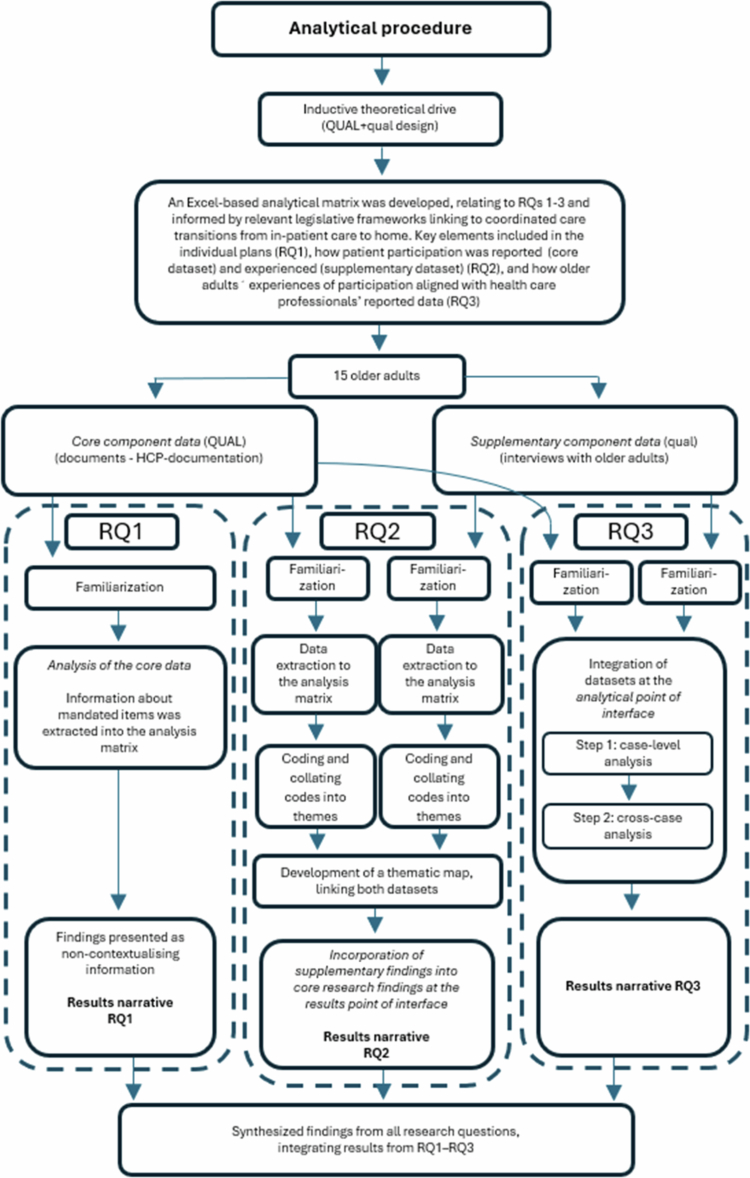
Overview of the (mixed-method) analytical procedure.

The analytical procedure was guided by the principles of a QUAL + qual design (Morse, 2010; Morse & Niehaus, 2016) and followed a theoretical thematic analysis as outlined by Braun & Clarke (2006); Braun & Clarke (2021). Relevant legislative frameworks were used to guide coding and focus the analysis on aspects of the data related to the study’s aims and research questions (Braun & Clarke, 2006). See Figure 1 for an overview of the analytical procedure. The analytical procedure was mainly performed by EI with support from KS. All authors discussed, reviewed, refined, and reorganised the findings through an iterative process until consensus was reached, as further described below.

### Legislative framework and analysis matrix

To operationalise the deductive orientation, the EI developed an Excel-based analytical matrix, informed by relevant legislative frameworks and aligning the study's aim with the three research questions (RQs 1–3) reviewed, discussed and approved by all authors. This matrix provided a structured, deductive approach informed by relevant legislative frameworks regulating healthcare and social care services concerning care transitions from in-patient care to a patient’s home.

All relevant legal documents - the Patient Act (SFS, 2014:821), the Patient Safety Act (SFS, 2010:659, 2010), the Health and Medical Services Act (SFS, 2017:30), the Social Services Act (SFS, 2001:453), and the Act on Coordination upon Discharge from In-Patient Care (SFS, 2017:612) - were systematically reviewed. Provisions specifying the required content of coordinated individual plans, provider responsibilities, and legal expectations regarding patient participation were colour-coded, extracted and operationalised into analytical categories. These categories were then organised in an Excel-based analysis matrix, with columns reflecting statutory requirements and rows capturing data from the core dataset (documents) and the supplementary dataset (interviews), providing a structured tool for data analysis.

The first section of the matrix corresponded to RQ1, addressing legislatively mandated content of coordinated individual plans (discharge plans and SIPs), including whether provider responsibilities were specified, what support was described as needed, the timing and location for establishing a SIP, and whether designated care contacts were included. The second section corresponded to RQ2, capturing how HCPs documented older adults' participation (core dataset) and how older adults experienced their participation (supplementary dataset). These data were collated across participants for each dataset within the analytical matrix. The final section addressed RQ3, focusing on the alignment between individual older adults' experiences and the corresponding HCPs' documented data, at both the case level and cross-case level.

### Data familiarisation and data extraction

The analytical procedure began with familiarisation with all data through repeated reading of both core (QUAL) and supplementary (qual) datasets (Step 1: familiarising yourself with your data).

This initial step of familiarisation and extraction focused on the explicit content of the data, i.e. what was written in the documents and stated during the interviews, without interpreting underlying assumptions or latent meanings (Braun & Clarke, 2006). Data extracts, pieces of raw data from both datasets were systematically extracted and plotted within the analytical matrix according to their relevance to RQs 1–3 by EI.

For RQ1, each legislatively mandated item was operationalised as a separate column in an Excel sheet, with each older adult's documents examined (only core dataset) in separate rows under each column. This was done by EI, and information was pooled for all participants and presented as non-thematic contextualisation information in the results narrative (Braun & Clarke, 2021) to clarify how individual plans aligned with legislative requirements (RQ1).

### Coding, thematic development and integration of datasets RQ2

For RQ2, the results of the analyses from the two datasets met at the *results point of interface,* meaning that the supplementary findings were incorporated into the narrative presentation of the core results (Morse, 2010). The process of coding, thematic development and dataset integration for RQ2 is described below.

#### Coding and thematic development

For RQ2, the analysis followed a systematic thematic approach as described by Braun and Clarke (2006). Extracted data from the two datasets were kept separate in their respective Excel sheets during coding and thematic development. The data was pooled within the analytical matrix for each dataset, and coded to identify meaningful features regarding how older adults' participation was reported by HCPs in healthcare and social care documents, SIPs and discharge plans (core dataset), and how it was experienced by older adults (supplementary dataset).

EI and KS independently reviewed and coded the extracted data at a semantic level. At this level, the analysis is focused on identifying and organising patterns based solely on the documents' content and participants' statements, without interpreting underlying assumptions or latent meanings (Braun & Clarke, 2006). EI and KS compared codes, discussed discrepancies, and refined coding through iterative discussions until consensus was reached (Step 2: generating initial codes).

**Figure 2.**
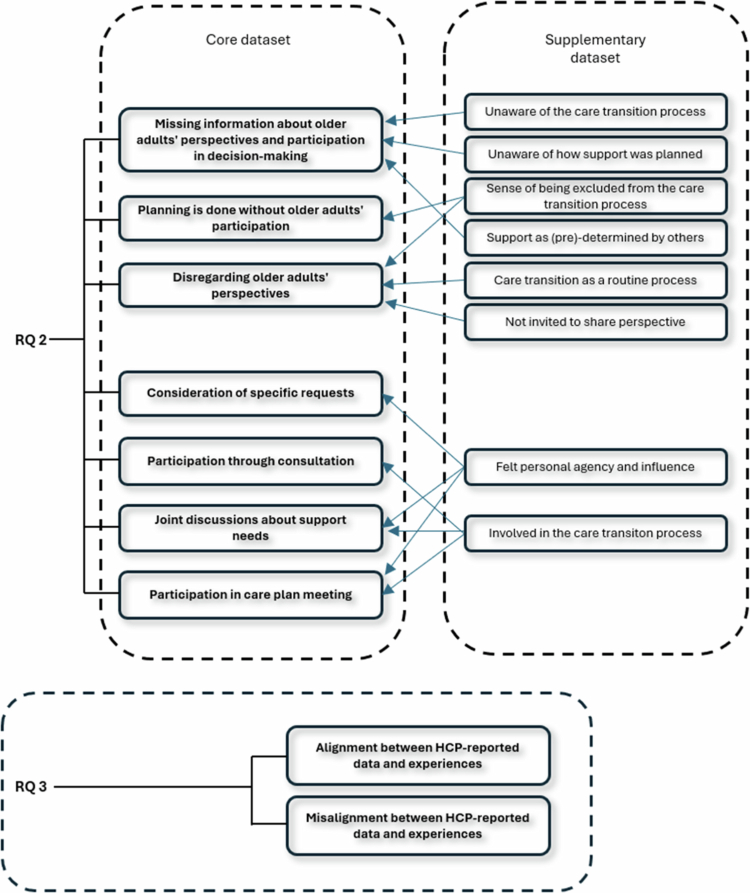
Thematic map of core and supplementary data components (RQs 2–3).

All codes were collated separately for each dataset and organised into potential themes by EI, discussed and revised among all authors in iterative discussions (Step 3: searching for themes), reviewed to ensure coherence in relation to both the coded extracts (Level 1) and the entire data set (Level 2) by EI (Step 4: reviewing themes). Each theme was refined, defined, and named through ongoing analytical discussions among all authors until consensus was reached, resulting in a thematic map (Step 5: Defining and naming themes), see Figure 2 for an overview.

#### Incorporation of datasets

The incorporation the supplementary datasets into the core dataset in the results narrative for RQ2 represents the final step of the thematic analysis (Step 6: Producing the report). As the analytical procedure followed the principles of a QUAL + qual design (Morse, 2010; Morse & Cheek, 2014; Morse & Niehaus, 2016), the datasets met at the *results point of interface*, meaning the core dataset provided the foundation for the results narrative into which supplementary data were incorporated at strategic points to illustrate aspects of older adults' experiences that were not accessible from the core documents alone. Each instance of incorporation was evaluated based on whether it corroborated the core data, provided additional descriptive detail or explanation, or highlighted discrepancies or nuances in older adults’ experiences (Morse & Niehaus, 2016).

### Coding, thematic development and integration of datasets RQ3

For RQ3, the core (documents) and supplementary (interviews) datasets were integrated during the analysis. The data were initially analysed at the level of individual cases (case-level) and then cross-case analysed (cross-case analysis), this was initially done by EI, drawing on principles from case-study design (Morse, 2010). Core data (documents) and supplementary data (interviews) were first analysed on a case-by-case basis within the analysis matrix, focusing on identifying patterns and characteristics within each case and how older adults' experiences of participation (supplementary dataset) aligned with HCPs' documentation (core dataset).

After the within-case analysis, findings were systematically compared across cases (cross-case analysis) to identify similarities and differences in how older adults’ experiences of participation aligned with HCP-reported data. Through this process, iterative discussions among all authors led to the development of two themes (see Figure 2 for an overview).

### Ethical approval and consent to participate

The study was approved by the Swedish Ethical Review Authority (Registration number 2020–01219), based on the Swedish national law, *Lag (2003:460) om etikprövning av forskning som avser människor* in Sweden (SFS, 2003:460). The Swedish Ethical Review Authority is an independent government agency responsible for ethical vetting of research involving humans in Sweden. [Redacted]. The study was conducted in accordance with the principles of the Declaration of Helsinki (World Medical Association, 2024). All older adults provided written informed consent to participate in the study. The consents were obtained by the first author in hand when the interviews were performed face to face and by mail when the interviews were performed by telephone. The consents were further required for the confidentiality assessments that needed to be performed by the healthcare providers before any data could be disclosed to the first author. As the data in this study were considered sensitive personal data, containing health information, it was essential to verify that there was legal support for their disclosure and that releasing the data posed no risk of harm or detriment to the older adults or their next of kin. The provided data was not anonymized. To protect participant anonymity during analysis and presentation of results, the first author assigned a number to each participant's dataset. All data, audio recordings, transcribed materials and hard copies of medical records were stored and processed in accordance with current legislation, i.e., the General Data Protection Regulation (2016/679) (General Data Protection Regulation, 2016).

## Results narrative

This study aimed to explore and describe how coordinated care transitions aligned with legislation, how individual plans aligned with legislative requirements, and how compliance with legislation concerning patient participation was noted in HCPs' documentation and reported and experienced by older adults when discharged from in-patient care to home.

Overall, our study revealed that there were inconsistencies in the documentation of discharge plans and SIPs, with a frequent lack of key components that should be included (RQ1). The HCP-reported participation of older adults in the care transition also varied. The documentation revealed that information regarding older adults' participation was frequently inadequate or absent, with records often indicating their exclusion or failing to capture their perspectives. However, the documentation also revealed that the care transitions often seemed to be well-coordinated among HCPs and sometimes were in compliance with legislation (RQ2). The results further revealed that there was both alignment and misalignment between HCP-reported and experienced participation or non-participation of older adults (RQ3). For an overview of the thematic map, see Figure 2. In the following presentation, numbers have been used to refer to each respective participant.

### The documents' alignment with legislative requirements (RQ1)

Out of the 15 older adults included in the study, nine had a documented individual plan. Of these, eight had a discharge plan (no. 1, 5, 10, 12, 13, 14, 15, 16), and one had a 'SIP' (no. 11), a coordinated individual plan documented in their records. In six cases, no individual plans were documented, in these cases, information about planned support appeared in other parts of the medical or social records. See Table I for an overview of the individual plans established.

**Table I:** Overview of the individual plans established.

Type of plan	Participant
Discharge plan	1, 5, 10, 12, 13, 14, 15, 16
SIP	11
**Reasons for not establishing an individual plan**	
No additional need for support	2, 3, 8
Planned support communicated verbally	6
Additional support was planned without any individual plan being established	4, 17

Discharge plans were missing in several cases, no plan was documented for older adults discharged without additional support needs (no. 2, 3, 8), when planned support was communicated verbally (no. 6), and when additional support was required upon discharge (no. 4, 17). Documentation from HCPs included notes on coordination of support, separate from the individual plans (cases no. 1, 2, 3, 4, 5, 6, 10, 12). Five out of eight discharge plans included information about a designated care contact (no. 1, 5, 10, 12, 14). The SIP (no. 11) did not include a designated care contact.

Information regarding the timing and location for establishing a SIP after discharge was lacking in the individual plans, and only one out of 15 older adults had a SIP during hospitalisation. For four out of 15 older adults, the need for a SIP was considered by and between HCPs, and only sometimes the older adult was included according to the documentation. Generally, when and where a SIP should be established was not described in the documents or discharge plans the older adults received. The one SIP identified lacked a specified time for follow-up of the SIP itself. The analysis of the documents revealed that one discharge plan (no. 16) and one SIP (no. 11) included a detailed summary encompassing all the HCPs involved regarding planned support and follow-ups after being discharged.

### HCP-reported participation of older adults and older adults' experiences (RQ2)

The analysis revealed key findings concerning the coordinated care transition process, compliance with legislation, and older adults’ participation. These findings were categorised into six sub-themes: missing information about older adults' perspectives and participation; missing information regarding decision-making; planning without older adults' involvement; disregarding older adults' perspectives; consideration of specific requests; and involvement through consultation.

Overall, the results showed variation in how older adults’ participation was documented by HCPs and experienced by older adults. Both the analysis of the documents (core dataset) and the interviews (supplementary dataset) indicated substantial gaps in meeting legislative requirements related to older adults’ participation.

In the following presentation of the results for RQ2, the findings from the separate analyses of the core data (documents) and the supplementary data (interviews) are integrated at the point of interface. That is, the findings from the analysis of the supplementary data are integrated into the narrative presentation of the core results, providing additional perspective and descriptive details at relevant points in the results narrative for RQ2.

#### Missing information about older adults’ perspectives and participation in decision-making

The documents rarely included older adults' perspectives on planning and participation in decision-making during their care transitions, particularly regarding support from municipal healthcare, paramedicine and social care services.

#### *Has home healthcare, personal alarm, and home help services, no new interventions.* (Core data component, no. 6)

Older adults' perspectives were sometimes missing from discharge plans but appeared in other documents. The documents showed that HCPs interacted with older adults through post-care information, joint home visits, and included them in discussions of social support needs. The documents frequently lacked clarity regarding who was responsible for decision-making, including when support was deemed unnecessary or when information about planning and applications for support was conflicting or vague. The interviews showed that older adults were often unaware of the care transition process, including how their support had been planned and the extent of their involvement. This finding aligned with the limited information found in the documents.

#### Planning without older adults' involvement

The analysis of the documents showed that support planning often excluded older adults, with crucial decisions made by HCPs and next of kin. The documents rarely described older adults as participating in discussions, planning activities, or decision-making processes, including decisions on care plan meetings, support, SIPs, or referrals. These occasions were often documented as being assessed and settled among HCPs without the involvement of older adults:

*The patient has recently been hospitalised. Wrote in Messenger to the municipal nurse, as the patient is on home healthcare, inquiring about the need for a SIP. The municipal nurse informed that they recently visited the patient, who seemed more spry than a week or so ago. […] Action: Will not convene a SIP meeting at this time, reconsider if needed [information and consultancy by phone with a patient representative by phone/other HC contact].* (Core data component, no. 4)

The documentation showed that detailed planning among HCPs, including coordination of support from municipal nurses, was usually recorded in separate documents and often not included in the written materials provided to the older adult (individual plans). The interviews provided additional perspectives, indicating that older adults frequently perceived themselves as not being asked for their viewpoints, felt uninvolved in discussions, and were mainly informed of decisions already made. They also described limited awareness of the coordinated care transition process and how support had been planned, which was sometimes perceived as support given as routine, happening without their involvement.

#### Disregarding older adults’ perspective

The core documents did not consistently reflect older adults' perspectives. Even when their requests or preferences were noted, the records sometimes indicated that these were not incorporated into care planning, for instance when support such as a SIP was planned despite the older adult declining it, or when expressed needs were recorded but not acted upon. Opportunities for older adults to apply for support were sometimes limited, for example when HCPs were absent from care planning conferences. Supplementary interview data provided additional perspectives, with older adults describing instances in which support was planned, and decisions were made without their input.

#### Consideration of specific requests

Some documents showed that older adults were actively involved in their care transitions and had their perspectives and specific requests documented and acted upon. This included wishes to return home without additional support, needs for municipal services, preferences for care plan meetings prior to discharge, requests for assistive devices, and statements of readiness to return home. The supplementary interview data provided additional perspectives, with some older adults describing experiences of having authority and influence over their care transitions, including control over decisions and the ability to impact the support they received.

#### Involvement through consultation

The analysis of the documents showed that older adults were sometimes involved in decision-making when HCPs were uncertain about support needs. In these cases, discussions were initiated, and decisions were made in consultation with the older adults. The supplementary interview data provided additional perspectives, with older adults describing situations in which HCPs discussed their need for continued support, particularly when being uncertain or experiencing impaired health.

#### Joint discussions about support needs

The documents showed that older adults were actively engaged in conversations about their support needs, with their preferences noted and considered by HCPs. The supplementary interview data showed older adults describing involvement when they were informed about decisions or when HCPs regularly discussed discharge and support needs during hospitalisation, including being asked about additional support needs.

#### Involvement in care plan meeting

The documents showed that care plan meetings that were conducted depicted as a platform where older adults were actively involved in discussions about their need for support, though this was rare. These meetings provided opportunities for older adults to receive information, express their needs, and make informed decisions, with HCPs documenting that their perspectives were considered. In some cases, older adults were also involved in decision-making when HCPs initiated discussions due to uncertainty about support needs. The supplementary interview data added further perspectives, with some older adults describing participation in care plan meetings and experiences of being asked for their views and being able to influence the support they received.

### How individual older adults' experiences of participation aligned with HCP-reported data (RQ3)

Both datasets were integrated during the analysis for RQ3, at the a*nalytical point of interface.* The case-level and cross-case analysis revealed differences between the HCPs’ documentation of older adults' participation (core dataset) and older adults' reported experiences from the interviews (supplementary dataset)*.* The findings were organised into two themes: *Alignment between HCP-reported data and older adults' experiences* and *Misalignment between HCP-reported data and older adults' experiences*, see Figure 2.

#### Alignment between HCP-reported data and older adults' experiences

In several cases, the HCP documentation of older adults (core dataset - documents) participation corresponded with the older adults' reported experiences (supplementary dataset - interviews). For example, in cases no. 2, 3, and 8, both HCP documentation and interviews described that older adults declined additional support and independently decided to return home. In case no. 3, documentation stated that the older adult “wished to return home without additional support,” which aligned with the older adult's reported experiences and description of managing independently and declining further involvement of healthcare staff.

Similarly, alignment was seen when HCPs reported that an older adult denied having additional need for support, and the older adult reported experiences of being listened to and being able to decline additional support when asked (no. 6). In these aligned cases, individual plans were absent, but documentation showed that the coordination of support had taken place among municipal nurses and was described in their individual documentation respectively. Older adults were sometimes informed about this verbally.

In other aligned cases, participation was stated but not further detailed in the HCPs documentation. In these cases, the older adults had individual discharge plans and reported that they had participated, for example by being listened to and influencing decisions about support (cases no. 5, 10, 16). In case no. 10, the older adult described discussing and adjusting planned support, whereas this interaction was not described in the documentation.

In case no. 13, both datasets described the older adult as present at care plan meeting and of being involved in planning. However, the interview also included the older adult's account of reported confusion about the discharge and limited information about planned support:

*Of course, I was a little confused. But the meeting was great, I have to say. It was, I think, a little chaotic. Because the day after, this coordinator I still don’t know the name of came by and said ‘I guess you’re going home.’ ‘Yeah, but…’ I said, ‘…it’s not up to me when I’m going home, right?’ Because I’d asked and they’d said ‘Yeah, the occupational therapist and physiotherapist will make an assessment of what you are capable of and so on.’ Yeah, no, I guess I can’t assess that. Yeah, and I guess she didn’t know what to say, so there were some little things like that, that I found a bit confusing at the […] department. I mean, the staff was just divine. They did absolutely everything they could. But it did feel like there was something that could be done about the organisation and structure there, I can say that much.* (Supplementary data component, no. 13)

In case no. 12 and no. 17, alignment was found where HCPs documentation noted older adults' views but also stated that these views were not incorporated into planning. Older adults described similar experiences of their perspectives being recorded but not acted upon. For example, in case no. 12 the HCPs documentation described the older adult’s view but noted that it was not incorporated into the planning. Consistent with the documentation, the older adult reported experiences of being misunderstood and disregarded. A similar pattern appeared in case no. 17, where documentation showed HCPs had noted the older adult's lack of interest in home healthcare in several places, but nevertheless planned for home healthcare together with next of kin, sidelining the older adult’s perspective:

*[xxx] is not interested in home healthcare at all. Thinks it works really well to go to the healthcare centre, doesn’t want to stop doing this and be stuck at home.* (Core component data, no. 17)

*Planning for home healthcare, it becomes clear that the patient can travel to the healthcare centre and may be able to do so in the future. The patient will be assessed by home healthcare after the first visit.* (Core component data, no. 17)

This aligned with the older adult’s own experience of feeling sidelined in the care transition process and not being asked about support needs, with only routine support being provided:

*No, I haven’t refused anything, I haven’t been asked about it anyway. [The wife says that ‘Well, they did ask you about that, or they called me, but I said no to home care, because I said that I’ll try to do it and we’ve never had home care.’] So there were no meetings with me? [Wife: No, there weren’t, they couldn’t call a meeting during Easter.* (Supplemental dataset, no. 17)

#### Misalignment between HCP-reported data and older adults' experiences

Several cases showed discrepancies between documentation and reported experiences. In case no. 1, HCP documentation described the older adult's participation in decision-making during care plan meeting, while the older adult reported not understanding that participation had taken place, or the information provided (no. 1). Hence, documentation described the older adult as having participated, but the older adult reported not understanding own participation or that decisions about support were being made.

Misalignment was also evident where documents were not always coordinated, and the documentation showed contrasting information about older adults' participation. In case no. 4, the documentation showed that the older adult had denied additional support needs and that planning activities were carried out between HCPs. At the same time, the documentation stated that the older adult had participated by denying additional support needs, while the coordination of the SIP and planned support occurred among HCPs without involving the older adult. In contrast, the older adult misunderstood what participation in the care transition process involved, was unaware of that participation had taken place, and described feeling excluded from decision-making (no. 4).

Misalignment was also found in case no. 11. The HCP documentation noted the older adult's participation and involvement at a care plan meeting for a SIP. The documentation stated that the older adult had requested support, while other entries showed that support was planned by HCPs and next of kin, and that the older adult was informed about the plan afterwards. Although the older adult participated in discussions during hospitalisation, further coordination was conducted between HCPs and next of kin without considering the documented perspectives and wishes of the older adult. The older adult described having expressed a different preference, which was recorded in the documentation but not incorporated into the planned support. The analysis also revealed instances of misalignment where HCPs documented older adults as having participated, even though the older adults reported not having had any influence over decisions.

Documentation often noted participation in discussions or care plan meetings, while the older adults themselves reported not having experienced participation or influence over the support provided. In case no. 14, the documentation described “joint decision-making,” whereas the older adult reported that the support had been determined by others. Another example of misalignment was found in case no. 15, where participation in discharge planning was documented, but the planning had been conducted by HCPs over the telephone. The older adult perceived the support as provided as routine, and did not recognise the documented participation.

## Discussion

This study demonstrates that older adults are frequently excluded from their own coordinated care transition processes, and that documentation is often incomplete or does not adequately address their participation. These overarching findings reveal significant shortcomings in both the implementation and documentation practices of coordinated care transitions, as required by legislation when older adults are discharged from in-patient care to their homes.

### Alignment of documentation of individual plans and legislative requirements (RQ 1)

Our study found significant gaps and inconsistencies in the documentation of SIPs and discharge plans for older adults discharged from in-patient care to home. The documentation revealed that older adults were frequently sidelined in the care transition process and that planning and decision-making were often performed by HCPs and next of kin, without the active involvement of the older adults. Previous studies have shown that there are critical gaps in care transitions from in-patient care to a patient’s home, often because HCPs prioritise care coordination over involving older adults in the discharge process (Bångsbo et al., 2017; Cam et al., 2023; Moore et al., 2017). According to current legislation, older adults should be involved in their care transitions, have their decision-making authority respected, and be able to receive and understand the information provided (SFS, 2014:821; SFS, 2001:453; SFS, 2017:30).

The study revealed that older adults' participation was sometimes not evident in the documents, and the older adults' own perspectives, preferences, or wishes were occasionally not reflected upon. This inconsistency in documentation suggests a lack of standardised procedures regarding documentation and highlights the need for all relevant information to be documented comprehensively, as well as for ensuring that information effectively reaches older adults. However, the lack of information described above could be due to HCPs believing they followed guidelines concerning the involvement of older adults in care transitions (Bångsbo et al., 2017). This could cause them to consider this an obvious fact, and therefore not necessary to include in documentation. This lack of essential information led to notable variation in the completeness and clarity of the documented individual plans. These findings align with previous research highlighting the challenges of care transitions resulting from continuity gaps, poor communication, and inadequate coordination among care providers and older adults (Beer et al., 2014; Cline, 2016; Kneck et al., 2019; Schoenborn et al., 2013). Patients also perceive these issues as significant barriers to achieving optimal discharge processes (Luo et al., 2024). This is in line with the findings of Vogel et al. (2024), who emphasise the need for improved communication and greater patient involvement prior to discharge.

Our study showed that although essential details were sometimes documented by HCPs, they were not always included in the coordinated documents provided to older adults. Although efforts were made to coordinate information among HCPs, critical details regarding older adults' understanding and preparedness for returning home (Ingvarsson et al., 2024) were frequently absent from the documents provided to the older adults themselves. This highlights the need for a more integrated approach to documentation to ensure that all pertinent information is conveyed to older adults, aligning with the recognised need for improved communication and greater patient involvement in discharge planning.

### Compliance with legislation concerning the participation of older adults (RQ 2)

Our study revealed significant gaps and inconsistencies in compliance with legislation in relation to older adults' documented participation. HCP-reported data suggested that documentation often lacked details regarding older adults' participation in planning and decision-making about support. For example, the documents often showed that support planning and coordination were conducted without involving the older adults, with decisions made solely by HCPs or in consultation with next of kin. This lack of older adults' participation was sometimes evident in the documents, and the older adults' perspectives, preferences, or wishes were not always reflected upon.

This echoes previous research indicating that older adults frequently feel excluded from the care transition process, with their concerns and preferences being overlooked (Liebzeit et al., 2023). Despite being invited to participate in planning and decision-making, older adults may struggle to influence their care and participate in decision-making (Jobe et al., 2019). Most older adults prefer to be involved in the care transition from in-patient care to home, albeit in differing ways (Gabrielsson-Järhult & Nilsen, 2016; Jobe et al., 2019; Nilsen et al., 2022; Schjødt et al., 2022) and to differing degrees (Nilsen et al., 2022).

Although there is growing recognition at the national level in Sweden of the importance of patient participation (SFS, 2014:821) and adoption of person-centred approaches (Swedish Government, 2020), our study found that practices often fall short. The inconsistency between documented participation and the actual involvement of older adults identified in our study underscores the challenge of translating legislative requirements into everyday practice.

Although some older adults were actively involved in their care transitions, documentation did not consistently reflect their participation. For instance, although some care-plan meetings documented active involvement of older adults, such cases were rare, and many reported being informed of decisions only after they had been made.

This highlights the ongoing challenge of translating legislative requirements into practice. Our results indicate a significant deficiency in involving older adults in their care transitions, in breach of current legislation—which emphasises their participation in such processes (SFS, 2017:612). These findings are supported by previous research suggesting that HCPs should address the needs reported by older adults in care transitions (Liebzeit et al., 2021), prioritise patient perspectives, and actively involve them in achieving optimal discharge processes (Yeh et al., 2024), as supported by patients' views (Luo et al., 2024). Actively documenting older adults' preferences and ensuring their involvement in planning and decision-making not only aligns with legislative expectations (SFS, 2014:821; SFS, 2001:453; SFS, 2017:30), but may also contribute to more effective discharge processes (Luo et al., 2024; Marsall et al., 2024).

### Alignment between HCP-reported data and older adults' experiences (RQ 3)

The study identified both alignment and misalignment between HCPs' documentation of older adults' participation and older adults' reported experiences of coordinated care transitions from in-patient care to home. Older adults' participation in care transitions was inconsistently captured in HCPs’ documentation, with some cases showing alignment between recorded data and experiences, while others demonstrated significant misalignment, leaving older adults feeling excluded or misunderstood.

Specifically, in cases such as no. 2, 3, and 8, HCP documentation accurately reflected older adults' decisions to decline additional support and manage independently, demonstrating clear alignment between documented data and patients' experiences. Similarly, cases no. 5, 10, and 16 showed alignment where older adults participated in planning and influenced decisions, even if HCP documentation provided limited detail.

However, alignment did not always indicate meaningful participation. For example, in cases no. 12 and 17, older adults' views were recorded but not incorporated into planning, reflecting alignment in documentation but not in older adults' ability to influence decisions or participate meaningfully.

Significant misalignment was found in cases no. 1, 4, 11, 14, and 15, where HCP documentation described participation, but older adults reported feeling excluded, misunderstood, or lacking influence over decisions. These discrepancies highlight that documentation alone does not guarantee real patient involvement, and that older adults' perspectives may be overlooked despite being formally noted.

The findings underscore the need for improved communication, structured involvement strategies, and documentation practices that accurately reflect older adults' actual experiences of participation. Ensuring that care plans incorporate older adults’ preferences and that older adults understand their role in planning and decision-making is crucial for high-quality, patient-centred care. These results align with previous findings emphasising the need for increased communication and the participation of patients in care coordination (Beer et al., 2014; Cline, 2016; Kneck et al., 2019; Luo et al., 2024; Schoenborn et al., 2013; Vogel et al., 2024), and studies highlighting the importance of approaches focused on patients' needs and preferences to improve healthcare delivery during care transitions (Marsall et al., 2024; Ulin et al., 2016).

## Strengths and limitations

This study employed a mixed-method QUAL + qual design, involving the simultaneous collection of two datasets: a core dataset of HCP documentation and a supplementary dataset of interviews conducted with older adults, which allowed us to broaden the scope of our analysis (Morse, 2010). The integration of the datasets occurred at multiple levels: in the results narrative for RQ2, and during the analysis for RQ3, adding depth and breadth to the data and enabling a thorough exploration of the research questions. The supplementary component provided perspectives that could only be interpreted in the context of the core dataset, facilitating a detailed examination of how coordinated care transitions aligned with current legislation (Morse & Niehaus, 2016). This methodological triangulation strengthened the credibility and confirmability of the findings through cross-validation from multiple sources (Lincoln & Guba, 1985).

A strength of the study is that data collection was systematic, and interviews were transcribed verbatim and checked against audio recordings, ensuring accuracy and dependability (Braun & Clarke, 2006). In addition, we incorporated quotes from our datasets and provided a detailed description of the analytical procedure, which further enhanced the credibility of the findings. Dependability was promoted through transparency in the research process, including a thorough description of the research methodology, sampling, and data collection (Lincoln & Guba, 1985).

The core dataset included only registered copies of healthcare and social care records from HCPs, meaning that some communication between HCPs and older adults may not have been captured. We acknowledge that the recording habits of HCPs may have introduced variability in documentation practices, potentially influencing our findings. Incomplete or inconsistent documentation may have led to underreporting or selective recording of patient participation, thereby compromising the accuracy and completeness of the dataset. Demsash et al. (2023) found that incomplete documentation accounted for about one-third of poor routine practices. Conversely, when HCPs possessed adequate knowledge and training, proper documentation was more likely, underscoring the influence of record-keeping practices on data completeness and reliability.

Individuals with cognitive impairment or dementia were excluded to ensure the reliability of self-reported data. Including these groups would have required alternative methodological and ethical approaches beyond the scope of this study. We acknowledge, however, that older adults with cognitive impairments or dementia face distinct challenges during care transitions, particularly regarding communication and involvement (Ashbourne et al., 2021). Therefore, the findings primarily reflect cognitively intact older adults, and the generalisability and the transferability to other populations or regions may be limited. The Swedish context, characterised by a tax-funded and decentralised healthcare system, also influences the transferability of the results to countries with different healthcare structures and legislation.

Transcripts and analyses were not returned to participants for validation, consistent with methodological discussions indicating that such member-checking does not always provide reliable verification at an aggregated level (Morse et al., 2002). Instead, accuracy was ensured during data collection, as advocated by Morse et al. (2002), by encouraging participants to elaborate on their responses and by using clarifying and probing questions to facilitate reflection on their experiences.

## Conclusion

The synthesised findings across all research questions (RQ1–RQ3) indicate that older adults are frequently excluded from and overlooked in their own coordinated care transition processes and that there are deficiencies in documentation. These findings highlight gaps between current practice and legislative requirements, underscoring the urgent need for standardised and inclusive documentation practices, as well as improved communication strategies. Such practices are essential to ensure more person-centred care transitions, in which older adults are genuinely involved in and well-informed about their care transitions, thereby aligning practice more closely with legal expectations and promoting meaningful patient participation, rather than overlooked.

## Implications for practice

The findings indicate that while some HCPs documentation corresponded with older adults’ reported experiences of participation, the alignment and misalignment between these perspectives in other cases underscores a persistent gap between documented participation and actual patient engagement. To address this, structured documentation and clear communication should be prioritised during care transitions, ensuring two-way communication and safeguarding older adults’ understanding of the information provided by HCPs. Older adults' perspectives and preferences also need to be systematically captured and considered in planning and decision-making. Enhancing documentation and improving HCP record-keeping practices can help ensure that participation is accurately reflected in clinical records. This makes older adults’ perspectives visible and supports the consistent incorporation of their voices in guiding care planning and discharge processes.

## Future research

Building on the current study, future research should include the perspectives of particularly vulnerable or underrepresented groups, such as older adults with cognitive impairments, dementia, or next of kin, by employing adapted methods to capture these viewpoints, including direct observations of interactions and care processes. This is essential for obtaining a more comprehensive and accurate understanding of patient participation in coordinated care transitions, extending beyond just vulnerable or underrepresented groups.

Additionally, studies should explore strategies to strengthen meaningful participation in coordinated care transitions, including the development and evaluation of interventions that improve communication, involvement in planning and decision-making, and documentation of older adults' perspectives. Future research could also evaluate how enhanced documentation practices influence patient participation and the overall quality of care transitions. Such research can provide practical, evidence-based insights into how person-centred care can be strengthened in coordinated care transitions, informing the design of clinical practices and interventions that support meaningful participation.

## Ethical statement

Our study was approved by the Swedish Ethical Review Authority (Registration number 2020–01219) and conducted in accordance with the principles of the Declaration of Helsinki. All participants provided written informed consent to participate in the study.

## Data Availability

To protect the privacy of the participants, the datasets generated in this study cannot be made publicly available, in accordance with current Swedish legislation, and per the Research Ethics Board’s guidelines. The audio recordings and interview transcripts are confidential as they contain sensitive personal data, and there is no consent from the participants for public availability of datasets.

## Appendix 1. Overview of documents included in the core data component and their origin.

**Table:**

Origin of documents	Key documents
**In-patient care**	“Nursing discharge summary,” “Discharge summary,” “Nursing status,” “Wound care,” “Nursing admission,” “Reception notes,” “Nursing notes,” “Occupational therapist and physiotherapist medical record notes,” 'Palliative care,” “Day-to-day notes,” “Care planning meeting notes,” “SIP notes,” and “Discharge plans”
**Primary care**	“Home visit notes,” “Telephone contact/other contact,” “Care planning meeting notes,” and “Notes without patient contact”
**Municipality**	Social care service managers: “Medical record notes,” “Investigation about support,” and “Decision regarding support”; Home help services: “Day-to-day notes on ongoing events”; Municipal nurse, physiotherapist, or occupational therapist: “Health and medical records” and “Medical record notes”
**Coordinated documents**	Coordinated individual plans, such as “discharge plans” and “coordinated individual plans” (SIPs)
